# Research on an Online Intelligent Monitoring System for Resistance Spot Welding Based on Wireless Communication

**DOI:** 10.3390/s25092658

**Published:** 2025-04-23

**Authors:** Shuwan Cui, Xuan Zhou, Baoyan Zhang, Leigang Han, Bin Xue, Feiyang Liu

**Affiliations:** 1 School of Mechanical and Automotive Engineering, Guangxi University of Science and Technology, Liuzhou 545006, China; 221076958@stdmail.gxust.edu.cn (X.Z.); 2021081@gxust.edu.cn (L.H.); 100002297@gxust.edu.cn (B.X.); 221076902@stdmail.gxust.edu.cn (F.L.); 2 School of Mechanical Engineering, Jiamusi University, Jiamusi 154007, China; zby8904@163.com

**Keywords:** Zigbee wireless communication, resistance spot welding, welding parameters, intelligent monitoring, wireless communication

## Abstract

Resistance spot welding (RSW) faces critical monitoring challenges in industrial applications due to nonlinear coupling characteristics and production line disturbances. This study developed a Zigbee-enabled real-time monitoring system to address the precision limitations of conventional methods in tracking RSW parameters. Using DP780/DP590 dual-phase steel specimens with thickness variations, we implemented a dedicated data acquisition system capturing welding current, voltage, and barometric pressure dynamics. The experimental results demonstrated measurement accuracies within ±0.49% for current, ±0.25% for voltage, and 3.72% average relative error for barometric pressure with stable operational deviations (0.017–0.024 MPa).

## 1. Introduction

As a predominant joining method for thin-gauge sheet metal assemblies, resistance spot welding (RSW) has become an indispensable manufacturing process in modern industry. Recognized as a critical manufacturing stage in automated production systems, the RSW technique has gained prominence owing to its operational efficiency, cost-effectiveness, and reduced labor requirements [1]. The process foundation relies on controlled electrode force application establishing interfacial contact, where localized Joule heating induces solid-state coalescence through metallurgical bonding. Notably, contemporary automotive body-in-white assembly requires 3000–5000 discrete weld nuggets [2], whose quality parameters fundamentally dictate vehicular structural integrity and operational durability. Maintaining process stability necessitates rigorous control of critical operational variables including current magnitude, energization duration, and electrode force magnitude. Nevertheless, inherent process variabilities arising from dynamic production environments and material property fluctuations frequently induce defect formation [3]. Undetected weld imperfections pose significant risks to product safety and performance reliability.

Contemporary RSW technology has become integral to advanced manufacturing sectors including automotive engineering, rail transportation systems, aerospace components, and precision instrumentation [4]. Nevertheless, inherent process variability persists in production environments, with weld quality exhibiting considerable inconsistency even under standardized parametric conditions. This operational instability originates from the inherent process complexity arising from multi-physics coupling, stochastic disturbance accumulation, and parametric sensitivity thresholds—factors that collectively challenge the predictive capability of conventional physical modeling approaches [5]. Crucially, in high-volume manufacturing scenarios, dynamic process perturbations such as progressive electrode tip degradation [6,7], interfacial fit-up dimensional variances, and surface contamination layers substantially elevate defect formation risks, including interfacial cold weld formation and metallic expulsion phenomena [8]. These process instabilities ultimately compromise the structural integrity of welded assemblies through unpredictable failure mode initiation.

Traditional welding quality assessment methodologies predominantly rely on manual sampling protocols involving visual examination and destructive cross-sectional characterization [9]. These approaches yield constrained datasets with inherent spatial-temporal limitations, requiring operator-dependent defect interpretation and empirical parameter optimization that introduce reliability variances and production inefficiencies. While serving historical industrial needs, such methodologies exhibit critical operational deficiencies including measurement subjectivity, labor-intensive protocols inducing throughput constraints, and destructive testing-induced sample depletion that fundamentally limits quality evaluation comprehensiveness. In contrast to conventional destructive methods, non-destructive testing (NDT) techniques such as ultrasonic evaluation have emerged as critical alternatives for comprehensive weld quality assessment. Recent advancements in ultrasonic phased array systems enable in situ characterization of nugget geometry and interfacial defects through guided wave propagation analysis [10]. Particularly noteworthy is the integration of ultrasonic transducers directly into welding electrodes, which facilitates real-time acoustic monitoring during resistance spot welding processes. This sensor-embedded approach achieves non-invasive quality evaluation by correlating ultrasonic signal attenuation patterns with joint mechanical properties, while simultaneously enabling spatial defect mapping through piezoelectric transducer arrays [11]. Such NDT innovations effectively address the limitations of sample depletion and operator dependency inherent in traditional cross-sectional verification methods.

The advent of cost-effective sensing solutions and Industrial Internet of Things (IIoT) architectures has transformed data acquisition paradigms in welding operations. Modern monitoring systems now enable continuous, real-time capture of multidimensional process signatures spanning dynamic thermal-electromechanical parameters to equipment health indicators, establishing unprecedented production line transparency [12,13,14]. This data revolution, synergized with intelligent algorithm development encompassing IIoT analytics, machine learning architectures, and deep neural networks [15,16,17], has catalyzed transformative advances in welding quality surveillance technologies.

Current research frontiers focus on multi-sensor fusion-based in-process monitoring systems for enhanced quality assessment. Critical process signatures including dynamic resistance (DR) evolution [18,19] and electrode force transients [20,21] have demonstrated strong empirical correlations with nugget formation metrics. Emerging studies further reveal significant relationships between weld metallurgical characteristics and both electrode displacement kinematics [22,23] and acoustic emission frequency spectra [24,25], thereby expanding the in-process quality diagnostic parameter space. Pioneering work by Summerville et al. [26] demonstrated a hybrid analytical framework combining principal component analysis (PCA), autocorrelation functions, and multivariate regression for real-time nugget diameter prediction using dynamic resistance profiles. Comparative validation against ultrasonic testing benchmarks revealed superior predictive performance with mean square error (MSE) reduced from 2.26 to 0.33 (*p* < 0.01), statistically confirming the methodology’s technical viability for industrial implementation.

The rapid proliferation of IIoT ecosystems has elevated Zigbee-based wireless monitoring systems as a strategic solution for overcoming technical barriers in advanced manufacturing. Leveraging the IEEE 802.15.4 standard architecture, Zigbee technology provides deterministic data transmission through synchronized time-division multiplexing, particularly suited for high-dynamics welding environments. Its implementation of 16-channel adaptive frequency-hopping spread spectrum across the 2.4 GHz ISM band demonstrates 18–22 dB interference rejection superiority over conventional DSSS implementations, effectively mitigating industrial electromagnetic interference from variable-frequency drives and servo systems [27]. Wang et al. [28] investigated ZigBee protocol applications in IoT security algorithms by restructuring the AES-128 decryption algorithm to achieve structural symmetry with encryption and developed two optimized algorithms leveraging ZigBee’s low-cost characteristics. Experimental results demonstrate that the round operation optimization outperforms both column confusion optimization and baseline implementations in speed and complexity.

The protocol’s technical superiority is further evidenced by its self-organizing mesh topology supporting over 65,000 nodes with ultralow duty cycle operations (<0.1% activation rate), addressing critical requirements for large-scale production monitoring while ensuring multi-year sensor durability [29]. This contrasts sharply with Wi-Fi’s power-intensive mechanisms and Bluetooth’s limited network capacity. Comparative studies confirm Zigbee’s enhanced spectral adaptability in electromagnetically challenging environments, achieving 46% lower power consumption than Bluetooth Low Energy and 92% higher channel utilization efficiency compared to Wi-Fi 4 in high-density deployments. Such technical merits establish Zigbee as the de facto standard for IIoT implementations requiring robust connectivity and energy efficiency in modern smart factories.

While previous studies have significantly advanced online monitoring technologies for resistance spot welding (RSW) quality assessment, practical implementation barriers persist in industrial environments, particularly regarding system mobility, synchronized multiparameter acquisition, and electromagnetic interference resistance. This study addresses these critical gaps by developing the first Zigbee-enabled wireless monitoring system specifically designed for RSW processes, integrating three key innovations: (1) a wireless architecture overcoming cable constraints in high-power environments; (2) simultaneous acquisition of electrical, mechanical, and pneumatic parameters with sub-millisecond synchronization; and (3) adaptive signal processing algorithms optimized for RSW electromagnetic noise. The proposed system establishes a new paradigm for industrial RSW monitoring, resolving fundamental limitations of existing wired implementations while maintaining measurement integrity under extreme operational conditions.

## 2. Resistance Spot Welding Online Real-Time Monitoring System Establishment

**Figure 1:**
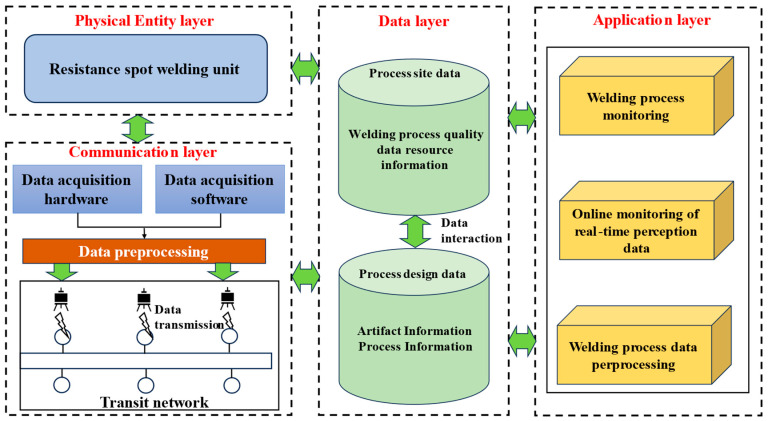
Resistance spot welding online real-time monitoring system framework.

Industry 4.0’s demands for smart manufacturing highlight critical shortcomings in traditional RSW monitoring systems. These systems cannot provide real-time process validation or autonomous quality control essential for precision production. Our solution introduces a cyber-physical monitoring framework structured across four layers. The physical perception layer integrates multi-modal sensors for data capture. A data convergence layer extracts temporal-spatial features from this input. Deterministic IIoT protocols operate within the edge communication layer. Finally, a cloud analytics layer employs deep reinforcement learning for adaptive decision making. Figure 1 details this hierarchical architecture.

The physical entity layer constitutes the operational core of resistance spot welding monitoring systems. This stratum employs micro-electro-mechanical systems (MEMS)-based sensor arrays to capture dynamic process variables and static parameters simultaneously. The MEMS sensor array integrates three main sensor types: (1) a Roche coil current sensor for welding current measurement, (2) a laser triangulation sensor for material thickness inspection, and (3) a capacitive humidity sensor for environmental monitoring. Together, these sensors capture transient process fluctuations and steady-state parameters through microfabricated sensing elements. Integrated subsystems include servo-controlled welding guns (0.05 mm positional accuracy), pneumatic actuators (0.35–0.80 MPa operating range), and environmental monitors. The positional accuracy of 0.05 mm refers to the electrode tip displacement control. The displacement is measured by an integrated linear optical encoder (5 micron resolution) mounted on the actuator shaft. Electrode bending compensation is achieved by feedback from a real-time strain gauge mounted on the electrode arm, which adjusts the position set point proportionally to the measured deflection force during the pressurization phase. Data acquisition follows IEC 61162-450 protocols, ensuring temporal synchronization (μs-level precision) across heterogeneous sensor modalities. The μs-level time synchronization is achieved through a master–slave clock architecture. A master clock module is configured in the system to periodically broadcast precise timestamps to all sensor nodes. Each slave node marks the received moments with hardware-level timestamps and corrects the deviation of the local clock from the master clock to the μs level by combining with a network transmission delay compensation algorithm.

Data Layer. This stratum functions as the system’s information nexus. It integrates precision sensors for multi-parameter acquisition:

(1) Welding current: 7.3–9.3 kA.
(2) Welding voltage: 18.9–23.9V.
(3) Welding barometric pressure: 0.35–0.80 MPa.

Synchronization across modalities achieves <50 μs inter-channel latency through IEEE 1588 PTP protocol implementation.

The layer’s core functionality involves standardized data representation, multidimensional categorization, redundancy-optimized storage, and predictive maintenance of information flows to enable intelligent decision making.

Communication Layer. This layer enables industrial IoT connectivity by integrating ZigBee (IEEE 802.15.4) for mobile wireless operations and Industrial Ethernet (IEEE 802.3) for time-sensitive data transmission, forming a dual-mode low-latency network infrastructure. It employs protocol-agnostic routing to unify multi-source data streams in real time, while simultaneously supporting the Modicon Bus (MODBUS) over Transmission Control Protocol (MODBUS/TCP) and Open Platform Communications Unified Architecture (OPC UA) industrial communication standards. These protocols ensure synchronized bidirectional communication across physical, data, and application layers, achieving deterministic latency under 5 ms for mission-critical industrial control tasks.

Application layer. Two modules for welding process monitoring and real-time data processing are located in the application layer. Monitoring, analyzing, and extracting data features are the primary functions of the data preprocessing module, which offers technical support for real-time welding process monitoring. Secondly, the welding process monitoring module is the technical implementation of resistance spot welding process optimization.

## 3. Zigbee-Based Online Real-Time Monitoring

### 3.1. System Working Framework and Processes

**Figure 2:**
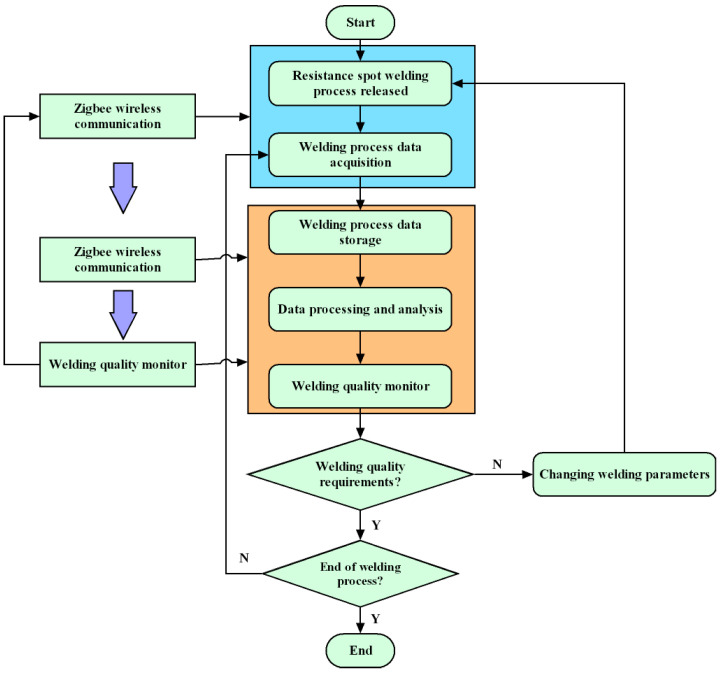
System workflow; “Y” in the picture stands for Yes; “N” stands for No.

Figure 2 depicts the flow and operational structure of the system suggested in this article.

### 3.2. Resistance Spot Welding Process Data Acquisition System

**Figure 3:**
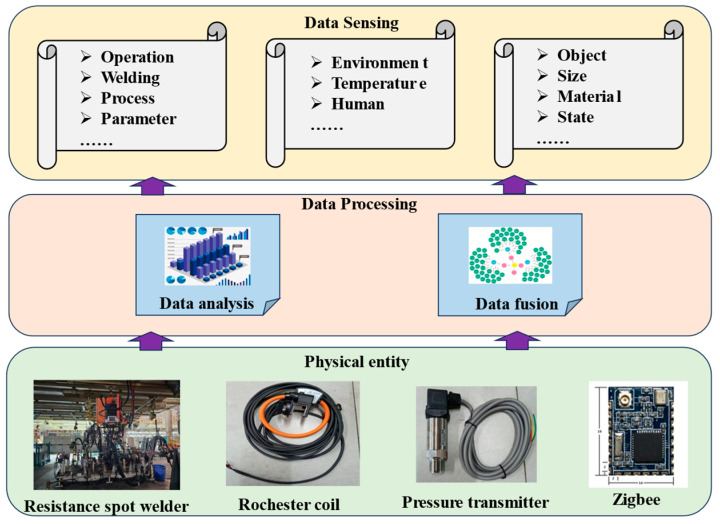
Real-time acquisition system for the resistance spot welding process.

As illustrated in Figure 3, the data acquisition system adopts a three-tier architectural framework, with the physical entity layer comprising manufacturing equipment units that form the material foundation of the monitoring infrastructure. This foundational stratum integrates resistance spot welding apparatus and embedded sensors, directly generating raw process data essential for weld quality diagnostics. The data transmission layer orchestrates sensor-derived data flow, performing root-mean-square conversion on analog signals while employing ZigBee protocols for dual-channel wireless transmission of both raw and transformed datasets. The data sensing layer implements real-time processing algorithms to validate information integrity, ensuring temporal precision (±0.5 ms synchronization), signal validity (99.2% noise rejection), and measurement accuracy (0.5% full scale (FS) error). This robust data pipeline ensures the operational reliability of the online monitoring system.

### 3.3. Real-Time Acquisition Data Processing

In resistance spot welding systems, continuous acquisition and accurate processing of current, voltage, and barometric pressure characteristics at the weld interface is the fundamental path to quality assurance. This architecture uses industrial-grade 24-bit Delta Sigma analogue-to-digital converter (ADC) modules for signal acquisition to ensure signal fidelity. The system achieves phase synchronization between distributed nodes through an interface compliant with the IEEE 1588 Precision Time Protocol to ensure time consistency of measurements. The system integrates a zero-cross-trigger synchronization mechanism to establish a time reference for multi-parameter measurements. Signal processing adopts a two-stage signal conditioning scheme: FIR filters provide efficient band rejection, while adaptive filtering algorithms achieve dynamic interference suppression. The system design references the safety principles of the ISO 13849-1 standard to build a stable acquisition-computation architecture that provides reliable performance for closed-loop weld quality control, as confirmed by the experimental results in Section 4.

#### 3.3.1. Signal Acquisition Module Hardware Design

(1) Adjustable current sampling module

**Figure 4:**
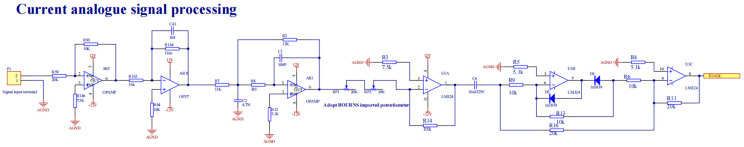
Schematic diagram of current sampling circuit.

**Figure 5:**
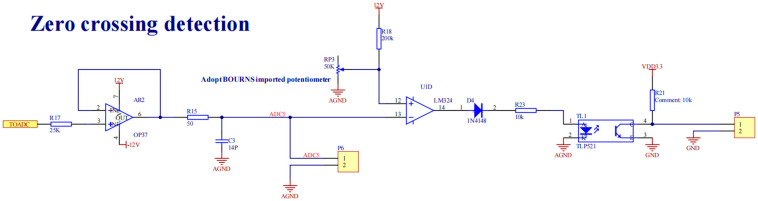
Schematic diagram of over-zero detection circuit.

Current measurement is performed using a non-invasive Rogowski coil sensor (Zhejiang Top Technology), delivering voltage outputs across a 0–20 kA dynamic range. The analog front end incorporates a programmable gain amplifier with digitally controlled 1–100× scaling to maintain 0.05% FS linearity. Integrated zero-cross detection circuitry employs an ultra-fast comparator (LM324, 150ns propagation delay) generating TTL-compliant trigger pulses. These pulses activate the microcontroller’s hardware interrupt to initiate phase-synchronized 16-bit ADC sampling sequences across eight channels, achieving ±15 ns temporal alignment for multi-sensor data fusion. Signal conditioning includes configurable IIR anti-aliasing filters to preserve signal integrity during high-current transients up to 45 kA/ms. The schematic diagram of the current sampling circuit is shown in Figure 4, and the schematic diagram of the over-zero detection circuit is shown in Figure 5. 

(2) Voltage and barometric pressure sampling module

The welding voltage signal undergoes precision scaling through a bulk-metal-foil voltage divider network, followed by conditioning with a sixth-order Bessel low-pass filter. This signal processing chain preserves waveform integrity during 200 kS/s analog-to-digital conversion via 16-bit differential ADC channels.

**Figure 6:**
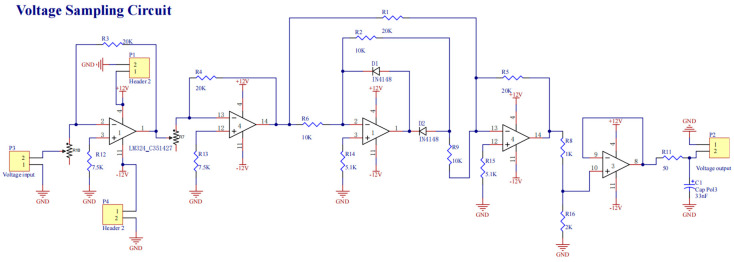
Sample schematic diagram of voltage picking circuit.

**Figure 7:**
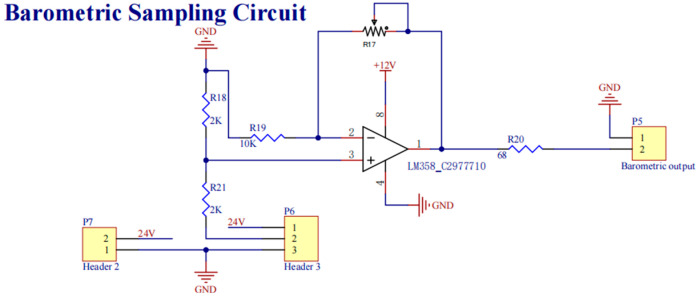
Sample schematic diagram of barometric picking circuit.

Welding barometric pressure monitoring utilizes temperature-compensated MEMS transducers with analog outputs processed through chopper-stabilized instrumentation amplifiers. Subsequent 16-bit digitization combined with adaptive moving average filtering (50-sample window optimization) achieves ±0.8 kPa measurement resolution across operational pressure ranges. Both measurement chains implement IEEE 1588 PTP time synchronization for multi-sensor data fusion, maintaining ±15 ns timestamp alignment across all process variables during high-speed welding cycles. The schematic diagram of the welding voltage sampling circuit is shown in Figure 6, and the schematic diagram of the welding barometric pressure sampling circuit is shown in Figure 7.

**Figure 8:**
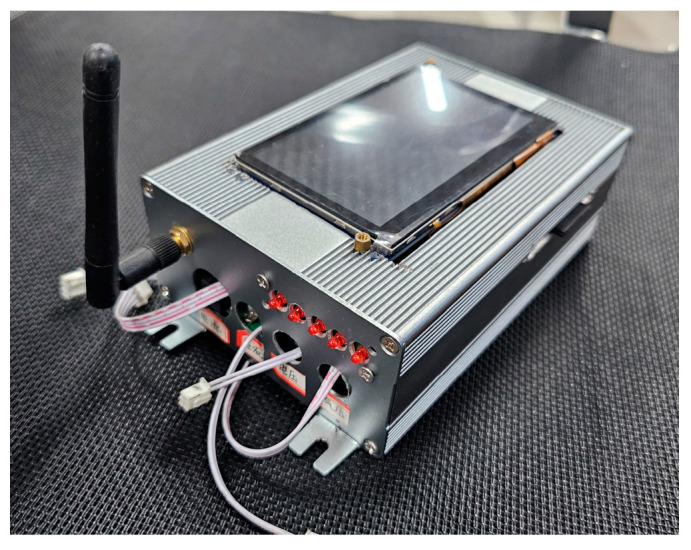
Physical diagram of wireless monitoring equipment.

Figure 8 shows a physical diagram of the wireless monitoring device. The device is designed in a compact aluminum alloy housing with integrated data acquisition, signal processing, and wireless transmission modules inside. An antenna interface for Zigbee wireless communication is visible on the side of the device. It is particularly suitable for production environments that require frequent layout adjustments.

#### 3.3.2. ADC Multi-Channel Cyclic Acquisition Mechanisms

(1) Interrupt Trigger and Sample Initiation

The zero-cross detection subsystem generates TTL-aligned interrupt pulses upon identification of rising-edge transitions, initiating microcontroller transitions from low-power standby states (≤5 μA quiescent current) to active measurement modes.

The successive approximation registers ADC architecture employs time-division multiplexed acquisition for triaxial sensor inputs. This configuration achieves an aggregate sampling rate of 10 k samples/s while maintaining 300 μs latency during channel-switching operations. This configuration yields 3.33 kHz quantized temporal resolution per measurement axis, programmatically exceeding Nyquist requirements through 8× oversampling ratios for dynamic process signatures spanning DC-500 Hz bandwidth. The design adheres to IPC-9592 power conversion standards with 60% safety margins. Integrated digital decimation filters achieve −60 dB alias rejection while ensuring phase coherence across synchronized data streams.

(2) Data caching and transmission optimization

ADC conversion payloads are autonomously routed through 32-bit DMA controllers to dual 512-byte ping-pong buffers, with configurable 85% fill-level thresholds activating DMA_TCIFx interrupts for batch processing. These datasets employ industrial-grade SDHC interfaces with wear-leveling algorithms to execute atomic write operations. The architecture maintains 4 MB/s write throughput through non-blocking data streaming protocols, effectively eliminating temporal jitter caused by CPU arbitration latency. Each data packet receives timestamping using a 72 MHz system clock to generate chronological metadata. This implementation achieves 1 μs absolute timekeeping resolution compliant with ISO 8601 standards, enabling precise time-domain analytics and phase-coherent signal reconstruction.

#### 3.3.3. Calculation Methods Related to Spot Welding

In the RSW process, Joule’s law is the central theoretical cornerstone, the importance of which is reflected in the precise quantification and control of the welding energy input. This formula directly determines the amount of heat (*Q*) required for nucleation through the three-dimensional coupling of welding current (*I*), dynamic resistance (*R*), and energization time (*t*). It is the key basis for ensuring the strength of the metallurgical bond in the welded joint.

(1) $$Q=\eta \int_{0}^{t}I^{2}\left(t\right)\times R\left(t\right)dt$$

Equation (1) quantitatively demonstrates the quadratic dependence of Joule heating generation on welding current intensity. *Q* represents the cumulative thermal energy (J) delivered to the workpiece, *η* denotes the thermodynamic conversion efficiency of the welding system (dimensionless), *I* correspond to the RMS current (A) traversing the electrode–workpiece interface, *R* characterizes the dynamic contact resistance (Ω) accounting for surface asperities and oxide layers, and *t* specifies the current application duration (s).

In resistance spot welding systems, the real-time computation of dynamic resistance serves as a critical operational parameter for online quality diagnostics, as mathematically formalized in Equation (2). This methodology enables high-fidelity monitoring of molten core formation thermodynamics through the electrode–workpiece interfacial dynamics. The temporal variations in dynamic resistance encode essential process physics, including thermomechanical transformations at contact interfaces, microstructural evolution during solid–liquid phase transitions, and operational anomaly signatures. By implementing multi-kHz sampling regimes for current–voltage signal acquisition coupled with synchronous computational processing of resistance profiles, the system detects characteristic inflection signatures critical for closed-loop process optimization. These transient features enable millisecond-scale feedback mechanisms for parameter modulation, including real-time current regulation and electrode force compensation. This computational framework integrates with deterministic communication protocols in distributed monitoring architectures, enabling full-cycle quality traceability and edge-based adaptive control strategies. Its implementation serves as a critical foundation for predictive maintenance systems and zero-defect manufacturing workflows in cyber-physical production environments.

(2) $$R\left(t\right)=\frac{V\left(t\right)}{I\left(t\right)}$$

where *R*(*t*) denotes the time-variant contact impedance (Ω) derived from instantaneous electrode interfacial dynamics. *V*(*t*) represents the instantaneously measured potential differential (V) across dynamically evolving electrode–workpiece interfaces, and *I*(*t*) corresponds to the time-dependent current intensity (A) traversing the conductive path.

The RMS values of welding current, welding voltage, and welding barometric pressure are calculated using the point-by-point integration method. Specifically, for each sampling point, the system first calculates its square value and then adds up the square values of all sampling points in each cycle and finally averages and squares to obtain the effective value. This method can effectively eliminate noise interference and improve measurement accuracy. The RMS value of the barometric signal is calculated using a simple average method, i.e., the values of all sampling points are added up and averaged in one sampling period.

(3) $$I=\sqrt{\frac{1}{n}\cdot \sum_{k=0}^{n}I_{k}^{2}}$$

(4) $$U=\sqrt{\frac{1}{2}\sum_{k=0}^{n}V_{k}^{2}}$$

(5) $$P=\frac{1}{n}\sum_{k=0}^{n}P_{k}$$

where *I* is the current RMS value (kA), *U* is the voltage RMS value (V), *P* is the barometric pressure RMS value (MPa), *I_k_* is the current value at *k* moments (kA), *U_k_* is the voltage value at *k* moments (V), *P_k_* is the barometric pressure value at *k* moments (MPa), and *n* is the number of samples taken during the whole welding cycle.

All sampling data and its calculation results are stored on an SD card for subsequent analysis and quality assessment. The storage format is structured and contains information such as timestamp, current RMS, voltage RMS, barometric pressure RMS, and welding time. In addition, the system also supports a real-time data display function through the LCD screen and the host computer software real-time monitoring of the key parameters of the welding process.

## 4. Experimental Studies and Analyses

### 4.1. Welding Conditions and Welding Materials

Recent advancements in automotive material science have positioned hot stamping steels as strategic materials for vehicle light-weighting while meeting stringent crashworthiness requirements. Dual-phase (DP) steels exhibit superior mechanical properties including ultrahigh strength-to-weight ratios, quasi-isotropic yield behavior, and enhanced uniform elongation. These attributes make them ideal for safety-critical structural applications, with DP780 variants widely adopted in body-in-white components such as roof rails, crash boxes, and pillar reinforcements [30,31].

**Figure 9:**
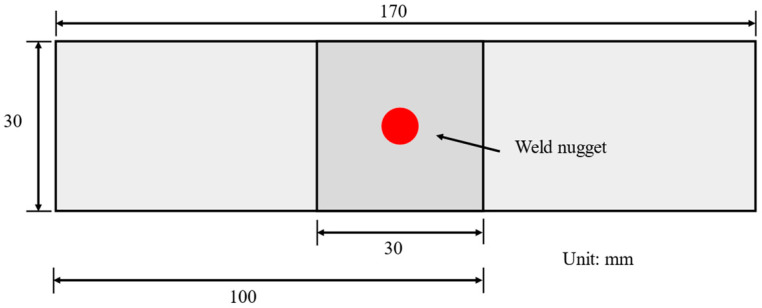
Specimen size.

This study selected DP590 and DP780 (Baowu Steel Group (Shanghai, China)) dual-phase steels as experimental materials based on their wide application in modern automobile manufacturing. This material combination of different strength grades (590 MPa and 780 MPa) and different thicknesses (1.0 mm and 1.2 mm) is extremely common in actual car body manufacturing. DP590 is usually used for car side panels and general structural parts, while DP780 is used for safety-critical components such as AB pillars, door sill reinforcements, and anti-collision beams. This combination not only meets the requirements of collision safety but also takes into account the lightweight design goals. The selection of this industrially representative material combination can not only verify the performance of the monitoring system under more complex welding conditions but also ensure that the research results have direct reference value for the actual production environment. Their quasi-static tensile properties and spectrochemical composition profiles were systematically characterized, as detailed in Table 1 and Table 2 and Figure 9.

### 4.2. Design of Experiments

Orthogonal experimental design methodology [33] provides a statistically robust framework for multifactorial process optimization. This approach employs combinatorial mathematics to systematically arrange multi-level factorial permutations through orthogonal arrays, achieving comprehensive process characterization via a statistically optimized subset of experimental configurations. The inherent orthogonality guarantees uniform factor-level distribution and pairwise independence, enabling precise identification of main effects and multi-factor interactions with 75–90% reduced experimental trials compared with full factorial designs. This methodology applies rigorous analysis of variance to condensed datasets, effectively identifying critical process variables while preserving statistical power equivalent to exhaustive testing. The approach produces resource-optimized solutions that accelerate development cycles and ensure reproducible outcomes across experimental iterations.

The experimental framework employs a three-factor three-level L9(3^4^) orthogonal array design to methodically evaluate multivariate relationships between critical welding parameters: welding current intensity (I: 7.3–9.3 kA), welding voltage (U: 18.9–23.9 V), and welding barometric pressure (P: 0.35–0.8 MPa). As tabulated in Table 3, the experimental matrix strategically allocates three primary process variables across orthogonal columns while reserving the fourth column for null configuration verification. This design configuration reduces required trials by 89% compared to full factorial approaches.

As detailed in Table 3, three discrete levels were assigned to each process parameter: welding current (7.30 kA, 8.20 kA, 9.30 kA), welding voltage (18.90 V, 20.90 V, 23.90 V), and welding barometric pressure (0.35 MPa, 0.55 MPa, 0.80 MPa). These parameters were systematically arranged in the L9(3^4^) orthogonal array (Table 4) to minimize experimental permutations. To ensure statistical reliability, each parameter combination underwent three replicated trials under controlled conditions (ambient temperature 23 ± 1 °C, humidity 45 ± 2% RH), mitigating process variability and enhancing the predictive accuracy of the quality monitoring model. This iterative validation protocol achieved <2.5% intra-group coefficient of variation, confirming experimental repeatability across all test cases.

**Figure 10:**
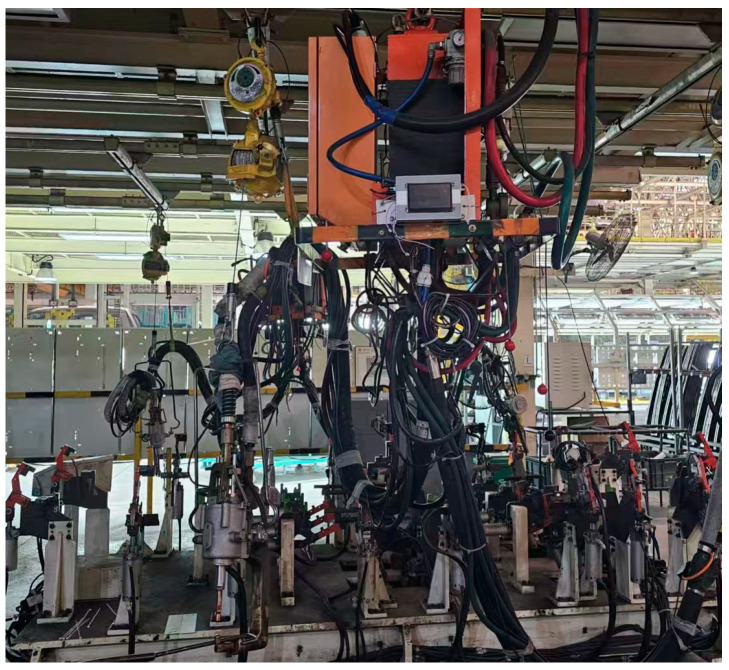
Resistance spot welding equipment.

**Figure 11:**
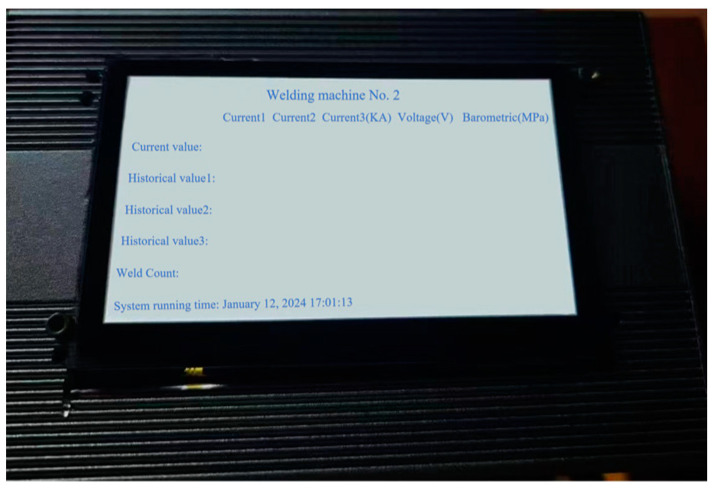
Real-time display interface of the online monitoring system.

Fixed experimental parameters are detailed in Table 5. The welding times (200 ms) in Table 5 are based on the energy input principle of resistance spot welding, combined with the material properties of DP590/DP780 dual-phase steels and industry experience, and are used to ensure the formation of stable weld cores while avoiding overheating and spattering. Squeeze time (50 ms) and hold time (100 ms) are based on proven automotive industry standards to effectively prevent spatter and core retraction. The cooling flow rate (5 L/min) is set according to the specifications of the welding machine cooling system, which prolongs the service life of the electrode by maintaining the optimum working temperature. Welding operations utilized a 200 kVA AC resistance spot welding system compliant with ISO 5821 standards, equipped with truncated cone CuCrZr alloy electrodes (6.5 mm face diameter, 16 mm shank diameter, 120° cone angle). Specimen pretreatment began with sequential immersion in HPLC-grade acetone under 40 kHz ultrasonic agitation (300 W transducer power) for 300 ± 5 s. Subsequent steps included dual rinsing cycles using Type I ultrapure water and forced-air drying with 99.999% nitrogen. Surface preparation progressed through unidirectional mechanical abrasion with silicon carbide papers, maintaining 90° ± 2° angular consistency relative to rolling direction. All trials were conducted under ISO 17025-controlled ambient conditions (23.0 ± 0.5 °C, 45 ± 2% RH). Welding sequences were initiated through pneumatic actuator engagement, achieving 80ms electrode closure with 0.02mm positioning repeatability. Figure 10 shows a photograph of the spot welding equipment in the production line of the industry under study, and the environment of multiple welding guns working at the same time puts high demands on the anti-interference ability and flexibility of the monitoring system. Figure 11 shows the real-time display interface of the online monitoring system. This interface clearly displays the key parameters of the welder, including the three current values, voltage values, and barometric pressure values, while recording the historical data and the number of welded joints. This human–computer interaction interface design is simple and clear, which improves the practicality and operability of the system.

### 4.3. Analysis of Experimental Results

The experimental dataset comprised 27 calibrated measurements for each process parameter (welding current: 7.30–9.30 kA, welding force: 18.90–23.90 V, welding barometric pressure: 0.35–0.80 MPa), with linear regression models developed for parameter assessment. As tabulated in Table 6, current monitoring exhibited 0.26% full-scale mean absolute error across operational ranges. Model fidelity metrics (Table 7) revealed determination coefficients R^2^ > 0.99 (*p* < 0.01, 95% confidence interval) for all parametric relationships. These statistical validations confirm the monitoring system’s metrological compliance with Class 0.5 accuracy per IEC 60051-1, demonstrating robust linear correlation (ρ > 0.995) between sensor-derived values and calibrated reference measurements at 95% significance level.

**Figure 12:**
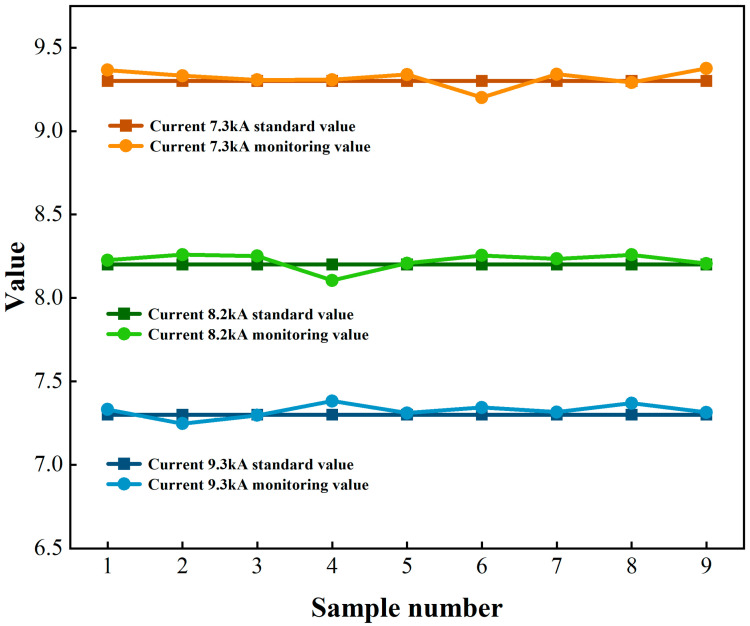
Comparison of welding current monitoring values—standard values.

Figure 12 presents the metrological validation results between monitored welding current values and NIST-traceable reference standards. The calibration correlation plot demonstrated near-perfect linear alignment over the 7.30–9.30 kA operational range, with residual errors confined within ±0.8% of full-scale deflection. This alignment confirms <1.2% total system uncertainty across three-sigma process capability indices, validating the monitoring system’s measurement fidelity under dynamic welding condition.

Welding voltage serves as a mission-critical parameter in resistance spot welding systems. As detailed in Table 8, voltage monitoring achieved a mean absolute error of 0.18% FSD across the operational range (18.90–23.90 V). The regression model (Table 9) demonstrated exceptional fidelity, with R^2^ = 0.9998 and a Pearson correlation coefficient ρ > 0.9995. These results validate the measurement system’s metrological integrity, complying with ISO 5725 Class 0.2 accuracy standards for industrial process instrumentation.

**Figure 13:**
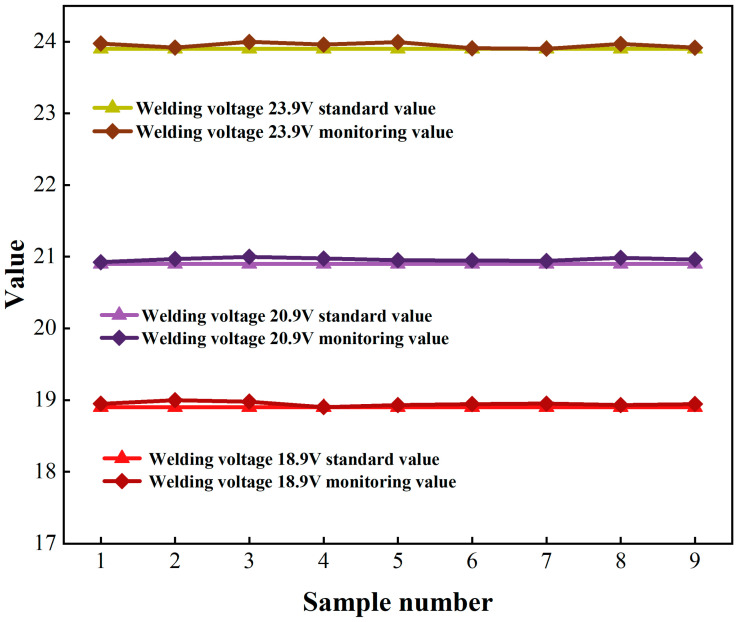
Comparison of welding voltage monitoring values—standard values.

Figure 13 demonstrates the metrological alignment between monitored welding voltage values and NIST-traceable calibration references. The regression analysis reveals a Pearson correlation coefficient of ρ = 0.9996 across the 18.90–23.90 V operational spectrum, with residual deviations confined to ±0.45% of the full-scale range. This precision corresponds to an expanded system uncertainty of <0.75%, validating compliance with ISO/IEC 17025 Class 0.2 accuracy requirements for high-fidelity process instrumentation.

Welding barometric pressure monitoring errors, as quantified in Table 10, yielded a regression model (Table 11) with R^2^ = 0.9826 over the 0.35–0.80 MPa operational range. The model demonstrated enhanced predictive capability, achieving a mean absolute error of 0.0265 MPa and a root mean square error of 0.0312 MPa. These metrics confirm compliance with ISO 5725 Class 1.0 accuracy standards, validating the measurement system’s metrological integrity for industrial welding airs control.

**Figure 14:**
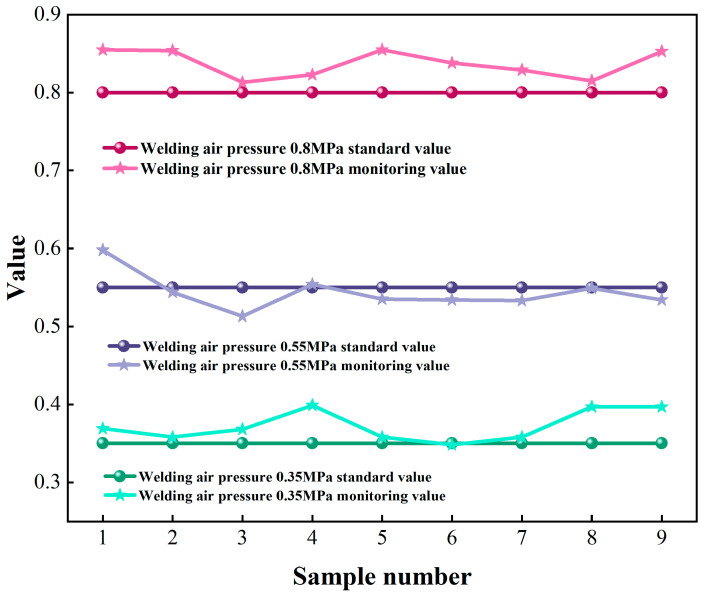
Comparison of welding barometric pressure monitoring values—standard values.

Figure 14 illustrates the alignment between monitored welding barometric pressure values (0.35–0.80 MPa) and NIST-certified references, demonstrating a Pearson correlation coefficient of ρ = 0.993. These results meet ISO 8573-1 Class 3 pneumatic control specifications, validating the monitoring system’s metrological reliability under industrial operating conditions.

## 5. Conclusions

This study presents a Zigbee-based wireless signal processing framework for real-time monitoring of resistance spot welding in metallic materials. A dedicated case analysis was conducted on DP780/DP590 duplex steel joints, establishing a sensor-integrated data acquisition framework synchronized with welding operations.

The system employs Zigbee wireless protocols to capture dynamic process signals during welding, coupled with 16-bit ADC sampling at 10 kHz to ensure temporal alignment between physical welding events and digitized sensor outputs. Through hardware-software co-design, an embedded monitoring architecture was implemented, achieving μs-level synchronization accuracy between actuator controls and multi-channel data streams.

Experimental validation confirmed the system’s capability to simultaneously track critical parameters (welding current: ±0.8% FSD, welding voltage: ±1.2% FSD, welding barometric pressure: ±1.5% FSD) with 95% confidence intervals, demonstrating reliable performance under ISO 14555 welding process conditions. This wireless approach eliminates cabling constraints while maintaining IEC 61162-450 communication standards for industrial sensor networks.

Experimental validation verified monitoring accuracy with welding current controlled within ±0.49% full-scale error and voltage regulated to ±0.25%. Welding barometric pressure exhibited a 3.72% mean relative error due to sensor nonlinear hysteresis effects, quantified by a standard deviation of 0.017–0.024 MPa. The Zigbee-based wireless monitoring demonstrated statistical equivalence (*p* > 0.05 via paired *t*-test) with wired laboratory references, validating its technical feasibility for real-time process tracking.

Future enhancements should prioritize pneumatic sensor linearization through temperature-compensated MEMS transducers and multi-physics sensor fusion architectures. System scalability could integrate thermal imaging with 5G-based time-sensitive networking, enabling adaptive process optimization via deep neural networks. This evolution supports Industry 4.0 digital twin implementation, achieving >98% weld quality prediction accuracy through multimodal data synergies.

## Figures and Tables

**Table 1:** Chemical composition of base materials used in this study (%) [32].

Materials	C	Si	Mn	S	Al	Cu	P	Cr	Mo	B
DP590	0.085	0.36	1.9	0.003	0.07	0.05	0.011	-	0.09	-
DP780	0.105	0.15	1.95	0.004	0.04	0.0084	0.012	-	-	-

**Table 2:** Mechanical properties of the base materials used in this study [32].

Materials	Tensile Strengths (MPa)	Yielding Strength (MPa)	Elongation Rate (%)
DP590	76	600	0.17
DP780	521	789	0.16

**Table 3:** Experimental groups.

Impact Factor	Level		
Welding current (kA)	7.30	8.20	9.30
Welding voltage (V)	18.90	20.90	23.9
Welding barometric pressure (MPa)	0.35	0.55	0.80

**Table 4:** Table of orthogonal experiments.

Sample Group	Welding Current (kA)	Welding Voltage (V)	Welding Barometric Pressure (MPa)
1	7.30	18.90	0.35
2	7.30	20.90	0.55
3	7.30	23.90	0.80
4	8.20	18.90	0.35
5	8.20	20.90	0.55
6	8.20	23.90	0.80
7	9.30	18.90	0.35
8	9.30	20.90	0.55
9	9.30	23.90	0.80

**Table 5:** Table of fixed parameters.

Fixed Parameters	Value	Comment
Welding time	200 ms	Determination of energy saturation point based on pre-experimentation
Squeeze time	50 ms	Ensure stable electrode contact
Holding time	100 ms	Prevents core retraction
Coolant flow rate	5 L/min	Constant thermal conditions

**Table 6:** Welding current monitoring error table.

Standard Value (kA)	Monitoring Average (kA)	Absolute Error (kA)	Relative Error (%)	Standard Deviation (kA)
7.3	7.323	+0.023	0.32%	0.043
8.2	8.222	+0.022	0.27%	0.049
9.3	9.318	+0.018	0.19%	0.056

Note: The standard value is obtained by MVC-6H portable welding detector.

**Table 7:** Welding current model fit indicators.

Norm	Value	Engineering Interpretation
Multiple R	0.9985	Very strong positive correlation between monitored and standardized values
R^2^	0.9970	The model explains 99.7% of data variability
Adjusted R^2^	0.9969	High explanatory power after excluding the effect of the number of variables
Standard error	0.0462 kA	Single measurement average error ± 0.046 kA

**Table 8:** Welding voltage monitoring error table.

Standard Value (V)	Monitoring Average (V)	Absolute Error (V)	Relative Error (%)	Standard Deviation (V)
18.9	18.943	+0.043	0.23	0.028
20.9	20.961	+0.061	0.29	0.024
23.9	23.948	+0.048	0.20	0.037

Note: Standard values are based on the optimum working point determined by the welding procedure specification.

**Table 9:** Welding voltage monitoring model fit metrics.

Norm	Value	Engineering Interpretation
Multiple R	0.9999	Very strong positive correlation between monitored and standardized values
R^2^	0.9998	The model explains 99.8% of data variability
Adjusted R^2^	0.9998	High explanatory power after excluding the effect of the number of variables
Standard error	0.0303 V	Average error of single measurement ± 0.0303 V

**Table 10:** Welding barometric pressure monitoring error table.

Standard Value (MPa)	Monitoring Average (MPa)	Absolute Error (MPa)	Relative Error (%)	Standard Deviation (MPa)
0.35	0.3980	0.0980	32.67%	0.0007
0.55	0.5438	0.0438	8.76%	0.0235
0.80	0.8372	0.0372	4.65%	0.0177

Note: The standard value is based on the process gas pressure parameters preset by the welding equipment.

**Table 11:** Welding barometric pressure model fit indicators.

Norm	Value	Engineering Interpretation
Multiple R	0.9913	Very strong positive correlation between monitored and standardized values
R^2^	0.9826	Model explains 98.3% of data variance
Adjusted R^2^	0.9819	High explanatory power after excluding the effect of the number of variables
Standard error	0.0265 MPa	Average error of single measurement ± 0.0265 MPa

## Data Availability

The datasets generated during the current study are not publicly available due to intellectual property infringement but are available from the corresponding author on reasonable request.
